# Enhancing the Quality of Buckwheat Noodles Through the Addition of Wheat Gluten and Wheat Flour

**DOI:** 10.3390/foods15173052

**Published:** 2026-08-28

**Authors:** Xiao Lei, Yuxuan Huang, Yulian Hu, Qiuling Tang, Xuefeng Zeng, Chen Liu, Xiuying Liu, Wei Zhang

**Affiliations:** 1 School of Food Science and Engineering, Wuhan Polytechnic University, Wuhan 430023, China; 2 National Engineering Research Center of Grain Storage and Logistics, Wuhan Polytechnic University, Wuhan 430023, China; 3 Key Laboratory for Deep Processing of Major Grain and Oil (Wuhan Polytechnic University), Ministry of Education, Wuhan 430023, China; 4 School of Liquor and Food Engineering, Guizhou University, Guiyang 550025, China

**Keywords:** buckwheat noodle, dough properties, rheological behavior, cooking quality, textural attributes

## Abstract

Common buckwheat and Tartary buckwheat are gluten-free pseudocereals with promising nutritional benefits; however, their poor dough functionality limits commercial noodle applications. This study evaluated six formulations: buckwheat flour with 12% added gluten, and blends consisting of 50% buckwheat (either common or Tartary) with 50% high-gluten wheat flour with an additional 6% or 12% gluten. The dough properties, rheological behaviors, moisture distributions, pasting properties of the flours, as well as the cooking performance and textural attributes of the resulting noodles, were systematically compared. Results showed that adding wheat flour and gluten markedly improved dough stability, particularly for Tartary buckwheat, which initially exhibited very weak structure. All doughs behaved as viscoelastic solids. For common buckwheat blends, the lower gluten level (6%) produced higher G′ and G″ than 12% level. Tartary buckwheat blends showed a positive correlation between gluten addition and viscoelastic moduli. Water was predominantly weakly bound, and Tartary buckwheat doughs retained more total bound water than common buckwheat doughs. Tartary buckwheat flours exhibited higher peak, final, and setback viscosities than common buckwheat blends. Tartary buckwheat noodles containing 50% wheat flour and 12% added gluten achieved superior cooking performance and textural attributes. These findings provide a practical strategy for producing high-quality buckwheat noodles.

## 1. Introduction

Buckwheat is a pseudocereal belonging to the genus *Fagopyrum* in the family *Polygonaceae* [1]. In 2024, global buckwheat output surpassed 1.97 million metric tons, based on estimates released by the Food and Agriculture Organization of the United Nations (FAO) [2]. The Russian Federation and the People’s Republic of China are the two largest producers, followed by Ukraine, Kazakhstan, and the United States. Among the genus *Fagopyrum*, common buckwheat (*Fagopyrum esculentum*) and Tartary buckwheat (*Fagopyrum tataricum*) are the two most widely cultivated species [3]. This is primarily due to the high tolerance of both species to diverse agroecological conditions [4,5].

Buckwheat grains are rich in starch, minerals, and vitamins [6]. These grains also contain considerable amounts of bioactive flavonoids, including rutin, quercetin, orientin, homoorientin, vitexin, and isovitexin [7,8]. In food processing, buckwheat is predominantly used in the form of flour, which retains these nutritional and bioactive components. Among the two cultivated species, Tartary buckwheat is of particular interest due to its substantially higher flavonoid content. The unique structural properties of buckwheat starch, together with the presence of these flavonoids, contribute to multiple health benefits. For instance, consumption of Tartary buckwheat flour has been reported to regulate intestinal NPC1L1 and ACAT2 and to increase fecal neutral sterol excretion—effects that are likely associated with its cholesterol-lowering properties [9]. Incorporating Tartary buckwheat flour into foods can enhance the abundance of beneficial gut bacteria, modulate intestinal microflora, and reduce the risk of metabolic diseases [10]. Furthermore, a rutin-rich Tartary buckwheat supplement demonstrated potential effects in reducing body weight, body fat percentage, and oxidative stress in a randomised, double-blind, placebo-controlled study [11].

Buckwheat is globally utilized in the preparation of various food products, including porridge, cakes, crisp biscuits, pancakes, and crepes [12]. However, its processing behavior differs substantially from that of wheat flour due to the absence of gluten-forming proteins, which impedes the production of wheat-like products [13]. Consequently, buckwheat flour is commonly blended with wheat or other flours prior to use. For example, Biduski et al. [14] demonstrated that replacing wheat flour with 20% buckwheat flour did not significantly compromise bread quality, whereas a 30% substitution level resulted in a dense and crumbly crumb structure. In contrast, Farzana et al. [15] reported that wheat flour enriched with 30% common buckwheat flour could still produce biscuits with acceptable sensory attributes.

Noodles are a staple food in Asia, and buckwheat noodles have long been popular in East Asia [16,17]. However, buckwheat proteins are inherently incapable of forming a gluten-like network [18], which severely limits their processing performance in noodle-making. Supplementation with exogenous gluten is a widely adopted strategy to overcome this technological bottleneck, yet it inevitably transforms the product into a gluten-containing system. Our primary objective is therefore to improve the processing adaptability of buckwheat for the general consumer market. In this study, varying levels of gluten and high-gluten wheat flour were incorporated into buckwheat flours to produce dried noodles. The quality attributes of common and Tartary buckwheat noodles formulated with different gluten and high-gluten wheat flours were systematically investigated, with the ultimate goal of promoting the utilization and development of buckwheat in staple food applications.

## 2. Materials and Methods

### 2.1. Materials

Common buckwheat flour (protein content 13.9%) was obtained from Gansu Dingxi Green Health Agricultural Products Processing Co., Ltd. (Dingxi, China). Tartary buckwheat flour (protein content 9.8%) was purchased from Weining Yalong Food Co., Ltd. (Bijie, China). Wheat gluten powder and high-gluten wheat flour (protein content 12.6%) were purchased from Henan Wanbang Chemical Technology Co., Ltd. (Zhenzhou, China) and Hebei Jinshahe Noodle Industry Group Co., Ltd. (Xingtai, China), respectively. Unless otherwise stated, all chemicals used were of analytical grade.

### 2.2. Formula of Buckwheat Noodle Flours

Buckwheat flour was blended with varying amounts of wheat flour and wheat gluten powder to formulate the noodle flours. Six noodle flour formulations were prepared using either common or Tartary buckwheat flour, as follows: CBF-12G and TBF-12G, common and Tartary buckwheat flour, respectively, supplemented with 12% gluten powder (*w*/*w*, dry basis relative to buckwheat flour); CBF-WF-12G and TBF-WF-12G, common and Tartary buckwheat flour, respectively, in a 50:50 (*w*/*w*) mixture with wheat flour, supplemented with 12% gluten powder (*w*/*w*, dry basis relative to the blend); CBF-WF-6G and TBF-WF-6G, common and Tartary buckwheat flour, respectively, in a 50:50 (*w*/*w*) mixture with wheat flour, supplemented with 6% gluten powder (*w*/*w*, dry basis relative to the blend).

### 2.3. Dough Property Analysis of Buckwheat Flour Blend

The dough properties of buckwheat flour blends were investigated using a Mixolab instrument (Chopin, Tripette & Renaud, Paris, France) according to AACC Method 54–60.01. The instrument was employed to determine water absorption (WA), dough development time (DDT), and dough stability time (DST), with data acquisition and processing performed using its integrated Chopin Mixolab 3.20 software package.

### 2.4. Rheological Property Analysis of Buckwheat Dough

A Kinexus pro+ rheometer (Malvern Instruments Ltd., Worcestershire, UK) was employed to assess the rheological properties of dough samples following a protocol according to Zhang et al. [19] with slight modification. The sample was positioned on the testing platform of the rheometer, utilizing a 4 cm plate with a gap set at 1.00 mm. Silicone oil was applied to seal the exposed edges of the dough to mitigate moisture loss during testing. All samples were allowed to rest on the plate for 2 min to achieve thermal equilibrium and facilitate the relaxation of induced stress. The experimental parameters were established as follows: strain at 0.1%, temperature maintained at 25 °C, and frequency scanning range from 0.10 to 10.00 Hz. Three duplicates were performed for each dough sample.

### 2.5. Low-Field Nuclear Magnetic Resonance (LF-NMR) Measurement of Buckwheat Dough

Proton distributions of buckwheat dough were measured using a NMI20-040V-I LF-NMR analyzer (Niumai Instruments, Suzhou, China). Dough was balanced at 25 °C for 20 min before measurement. The transverse relaxation (T_2_) curves were obtained using the Carr-Purcell-Meiboom-Gill (CPMG) pulse sequence. The main measurement parameters were as follows: receiver bandwidth = 200 kHz, repeat sampling wait time = 2000 ms, echo time = 0.2 ms, number of echos = 4000, and scan number = 8. The CPMG decay curves were fitted to a multi-exponential function model to obtain the transverse relaxation (T_2_) distribution spectra.

### 2.6. Pasting Property Determination of Buckwheat Noodle Flour

The pasting profiles of buckwheat noodle flour were determined using a Super-4 Rapid Visco Analyser (Newport Scientific Pvt. Ltd., Warriewood, Australia). The noodle flour (3.0 g, 14% moisture basis) was weighted into an aluminum canister, and 25 g of distilled water was added to attain a total sample weight of 28.0 g. The flour dispersion (12% *w*/*w*) was equilibrated at 50 °C for 1 min and then increased to 95 °C at a rate of 12 °C/min, held for 2.5 min, cooled to 50 °C at a rate of 12 °C/min, and held for 2 min. The paddle speed was set at 960 rpm for the first 10 s and then was reduced to 160 rpm throughout the rest of the experiment.

### 2.7. Preparation of Buckwheat Noodles

The noodle flour was poured into an HMJ-A35M1 dough mixer (Little Bear Electric Appliance Co., Ltd., Foshan, China). Water was added based on the optimum water absorption of the flour blends, as determined by the Mixolab. The mixture was kneaded for 10 min and the produced dough was then allowed to rest for 20 min. The rested dough was sheeted using an FKM sheeter (Yongkang Fukang Electric Appliance Co., Ltd., Jinhua, China) and subsequently cut into fresh noodles of 1 mm thickness, 2 mm width, and 20 cm length. The freshly made noodles were arranged on a drying rack and air-dried at room temperature and ambient humidity for 2 days to a moisture content of approximately 12.5%. The dried noodles were stored in plastic bags for later use.

### 2.8. Cooking Quality Measurement of Buckwheat Noodles

Cooking quality of buckwheat noodles was determined according to the method of Ning et al. [20]. Forty strands of intact dried noodles were cooked in boiling distilled water at a water-to-noodle ratio of 50:1 (*w*/*w*). The optimum cooking time was defined as the moment when the white core in the cross-section of the cooked noodles completely disappeared [20]. Another 40 strands of dried noodles were cooked under identical conditions for the same optimum cooking time. Water absorption during cooking was calculated as the percentage increase in sample weight relative to the initial dry noodle weight [21]. The cooked breakage rate was calculated as the mass ratio of broken noodles to total cooked noodles [20]. Cooking loss was defined as the percentage of solid substances leached into the cooking water, determined by evaporating the cooking water to dryness and expressing the residue mass as a percentage of the initial dry noodle mass [20].

### 2.9. Texture Analysis of Cooked Buckwheat Noodles

Textures of cooked buckwheat noodles were tested using the TA. Touch texture analyzer (Shanghai Baosheng Industrial Development Co., Ltd., Shanghai, China). Fifteen strands of dried noodles were boiled in 500 mL of water until the optimal cooking time and then immediately immersed in water at room temperature for 10 s. Subsequently, three of the cooked noodles were placed on a plate, and a TPA test was performed using a 36 mm cylinder probe according to the methods of Liu et al. [21] with slight modification. The test was conducted with following conditions: pre-test 1.0 mm/s, test speed 1.0 mm/s, post-test speed 1.0 mm/s, trigger force 5.0 g, and the compression ratio was 50%. The following texture parameters were recorded: hardness, springiness, chewiness, cohesiveness, and resilience. The above procedure was repeated on six independent cooked noodle samples for each flour formulation.

### 2.10. Statistical Analysis

Data are averages of at least triplicate observations and expressed as means ± standard deviations. An analysis of variance with a significance level of 5% was conducted and Duncan’s test was applied to determine differences between means using SPSS 19 (SPSS Inc., Chicago, IL, USA).

## 3. Results and Discussion

### 3.1. Dough Properties of Buckwheat Noodle Flour

The Mixolab characteristic values of buckwheat-based flours are shown in Table 1. For common buckwheat-based blends, adding wheat flour plus 12% gluten (CBF-WF-12G) gave the highest WA and longest DST, indicating excellent hydration capacity and dough tolerance, which is ideal for producing smooth, non-sticky noodle dough that resists breakdown during sheeting. The same common buckwheat blend with only 6% gluten (CBF-WF-6G) showed slightly lower WA but still good stability, suggesting that 6% gluten may be sufficient for noodle making. Studies have shown that the increase in buckwheat flour proportion in wheat-based composites generally elevates water absorption while reducing dough stability, primarily due to the dilution of the gluten network and the competitive water absorption by buckwheat proteins and fibers [22]. In contrast, Tartary buckwheat with 12% gluten but no wheat flour (TBF-12G) exhibits very short DDT and the lowest DST, indicating a weak, unstable dough unsuitable for mechanized noodle production. This inferior performance may be partially associated with the distinct physicochemical properties of Tartary buckwheat components, which may interfere with gluten network formation and dough cohesion. However, when Tartary buckwheat is combined with 50% wheat flour and either 6% or 12% added gluten (TBF-WF-6G and TBF-WF-12G), DST increases significantly to 5.37–5.49 min, demonstrating that the presence of wheat flour and sufficient gluten can overcome the inherent poor stability of Tartary buckwheat. Notably, water absorption for these Tartary blends remained relatively low, which may require recipe adjustments to achieve proper dough hydration. Overall, for both common and Tartary buckwheat, the combined addition of wheat flour and sufficient gluten (6% or 12%) can produce balanced and robust dough properties for high-quality buckwheat noodles.

### 3.2. Rheological Property of Buckwheat Dough

**Figure 1:**
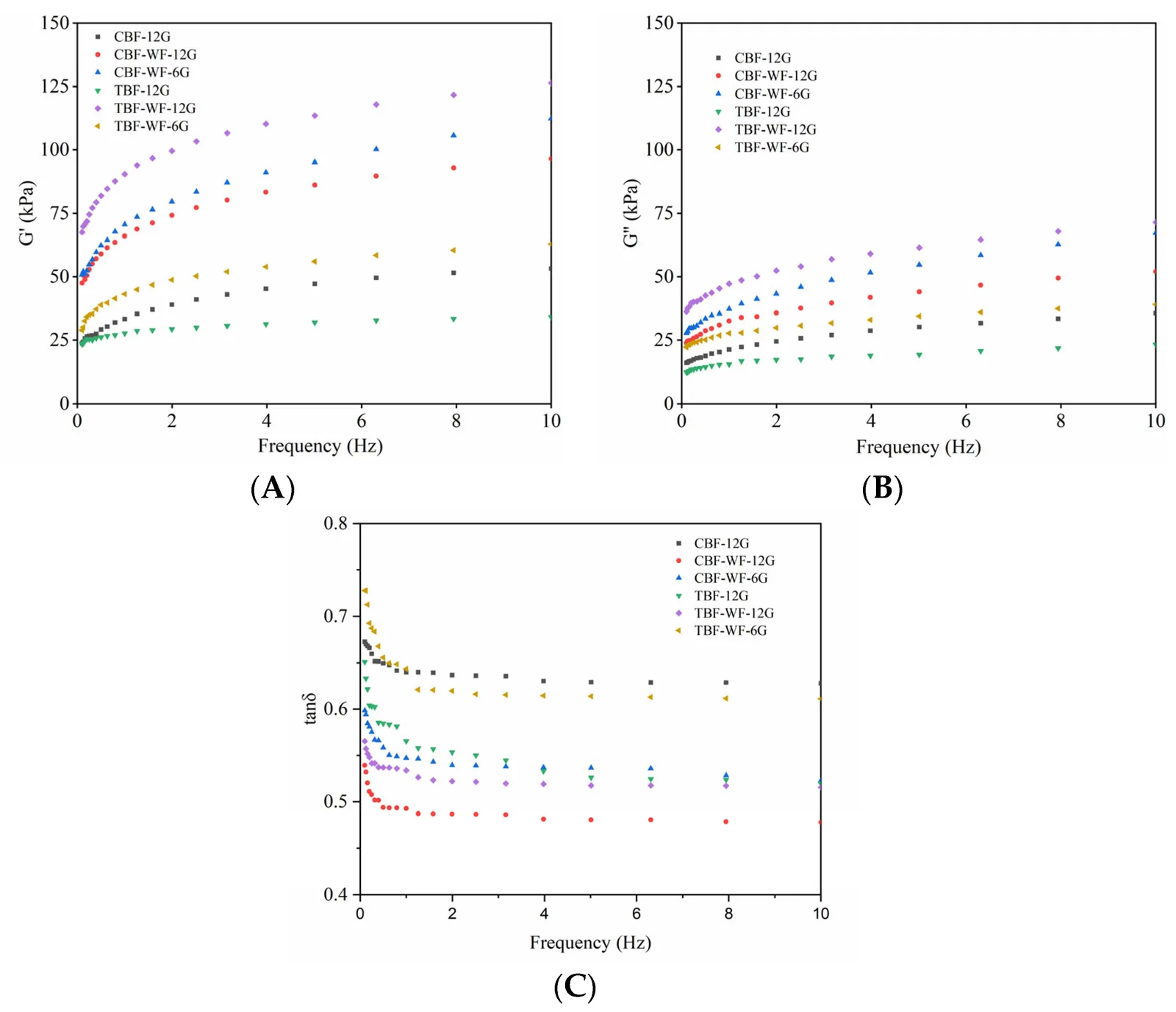
Storage modulus (G′), loss modulus (G″), and loss factor (tan δ) of doughs prepared with different buckwheat flour blends during frequency sweeping tests. (**A**) G’. (**B**) G″. (**C**) tan δ.

The storage modulus (G′) and loss modulus (G″) represent the elasticity and viscosity of the dough, respectively, while tan δ is the ratio of G″ to G′. The rheological properties of different buckwheat composite doughs were analyzed, and the results are presented in Figure 1. Over the entire frequency range, all doughs exhibited a typical viscoelastic solid-like behavior with G′ consistently higher than G″ (tan δ < 1) [23]. For common buckwheat-based blends, CBF-WF-12G and CBF-WF-6G showed higher G′ values than CBF-12G, suggesting that the combination of 50% wheat flour and added gluten powder may effectively enhance the dough network. This result is consistent with the Mixolab data. Notably, the G′ and G″ of CBF-WF-6G were higher than those of CBF-WF-12G, indicating that, for common buckwheat blends, excessive addition of wheat gluten powder does not necessarily lead to the expected increase in G′ and G″ values [24]. Compared with common buckwheat flour (without added high-gluten wheat flour), Tartary buckwheat flour exhibited considerably lower G′ and G″ values, indicating a weaker viscoelastic structure. The presence of 50% wheat flour in Tartary buckwheat flour significantly increased the G′ and G″ values of the resulting dough. Furthermore, for wheat flour-containing Tartary buckwheat flour, the G′ and G″ values of dough increased with elevated amount of wheat gluten. These findings suggest the dough network of Tartary buckwheat flour may be effectively enhanced by incorporating wheat flour and adding wheat gluten. However, direct microstructural evidence is required to conclusively establish the network-level changes underlying these rheological differences, and such investigation is planned in our subsequent studies.

### 3.3. Moisture Distribution of Buckwheat Dough

**Figure 2:**
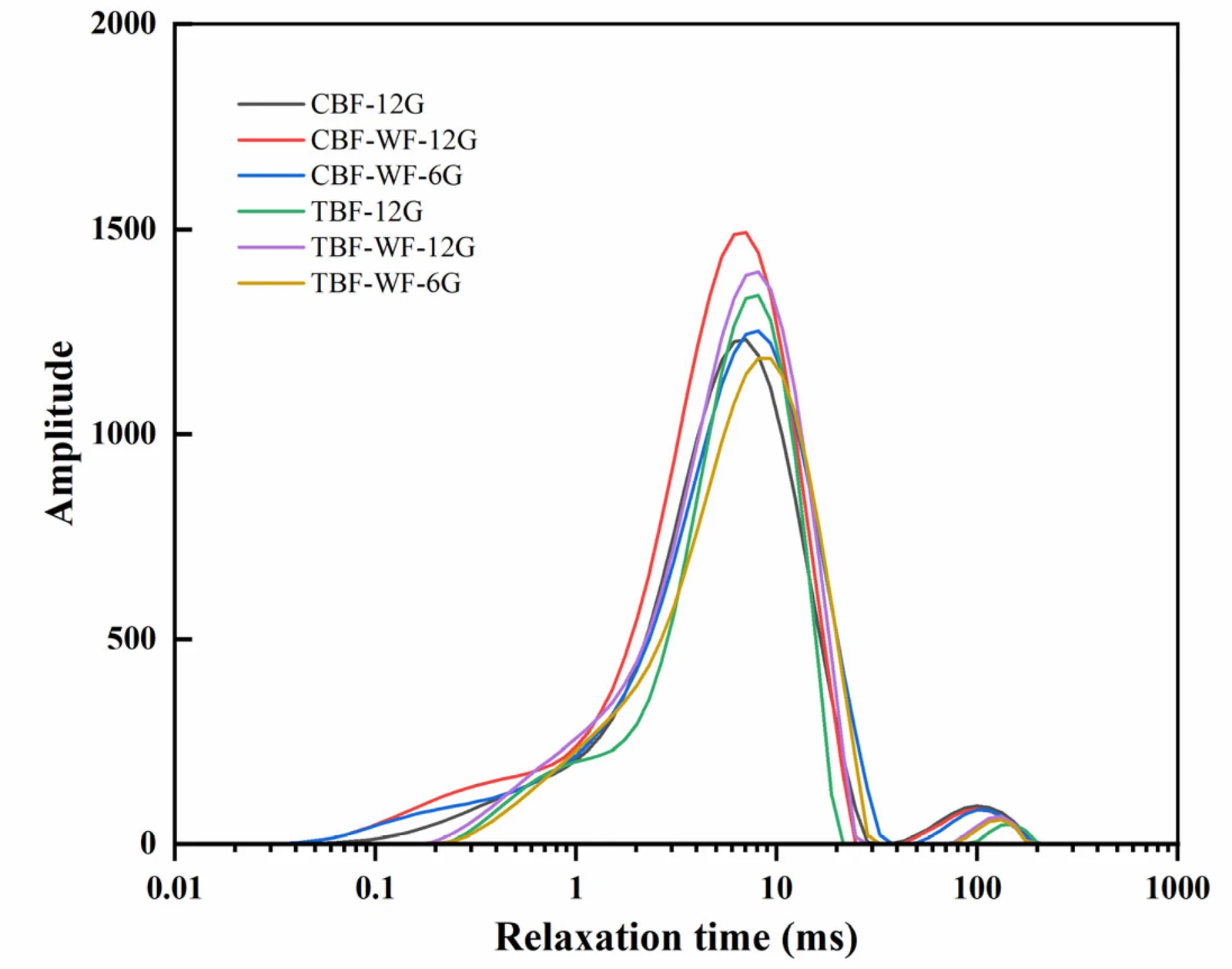
T_2_ inversion plots of doughs prepared with different buckwheat flour blends.

The moisture distribution in buckwheat noodle doughs was characterized by LF-NMR transverse relaxation time (T_2_) measurements. As shown in Figure 2 and Table 2, the majority of water across all formulations existed as weakly bound water, while the proportion of tightly bound water was consistently minimal. This indicates that water is primarily physically entrapped within the dough matrix. Among common buckwheat-based blends, adding 50% wheat flour plus gluten (CBF-WF-12G and CBF-WF-6G) slightly increased total bound water versus the wheat-free blend (CBF-12G), suggesting that a more continuous gluten network enhances water entrapment without altering binding strength distribution. Notably, all Tartary buckwheat doughs showed higher total bound water, but this increase was also mainly due to weakly bound water, with no substantial rise in the tightly bound component. As reported in similar systems [25], such water immobilization may be speculatively linked to weak interactions (e.g., hydrogen bonding); however, direct structural evidence (e.g., from Fourier Transform Infrared Spectroscopy and Scanning Electron Microscopy) is needed for confirmation. The superior water retention of Tartary buckwheat doughs does not necessarily translate into better viscoelasticity (Figure 1), which may also be correlated with flour composition. Unlike common buckwheat blends, adding 50% wheat flour plus gluten to Tartary buckwheat flour slightly increased free water content.

### 3.4. Pasting Property of Buckwheat Flour Blend

**Figure 3:**
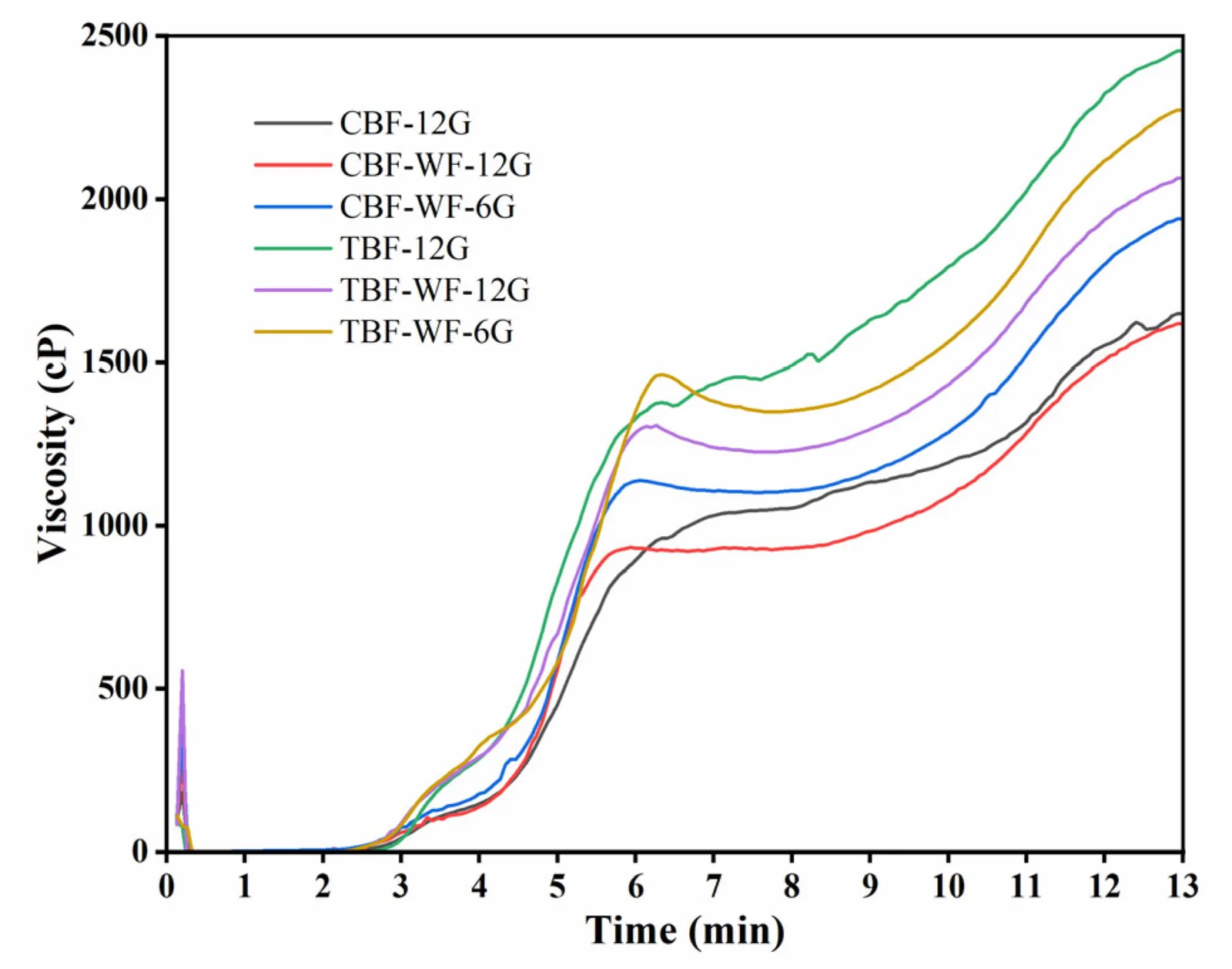
RVA curves of doughs prepared with different buckwheat flour blends.

The RVA profiles of noodle flours are shown in Figure 3, and the pasting behavior parameters are summarized in Table 3. Tartary buckwheat-based flours consistently exhibited substantially higher peak viscosity (PV), final viscosity (FV), and setback viscosity (SBV) than their common buckwheat-based counterparts, indicating greater swelling capacity, paste thickening ability, and retrogradation tendency [26]. Regarding shear stability, the Tartary buckwheat blends containing wheat flour (TBF-WF-12G and TBF-WF-6G) showed markedly higher breakdown viscosity (BDV) compared to other formulations, implying that the combination of Tartary buckwheat with wheat flour and added gluten produces a paste more susceptible to mechanical disintegration during cooking. In contrast, buckwheat pastes without wheat flour exhibited very low BDV, which may be attributed to either limited starch swelling or high resistance to shear during heating. Notably, the standard deviation of BDV was higher than those of the other viscosity parameters. In the RVA test, BDV is calculated by subtracting trough viscosity (TRV) from peak viscosity (PV); consequently, when PV and TRV are close, the resulting BDV tends to be low, yet its standard deviation may remain relatively high. Pasting temperatures varied among blends, with TBF-12G showing the lowest and TBF-WF-6G the highest, suggesting that the inclusion of wheat flour and gluten alters the pasting behavior of the blend. Overall, Tartary buckwheat flours generate thicker, more viscous pastes with strong setback, suggesting a higher retrogradation tendency.

### 3.5. Cooking Properties of Buckwheat Noodles

**Figure 4:**
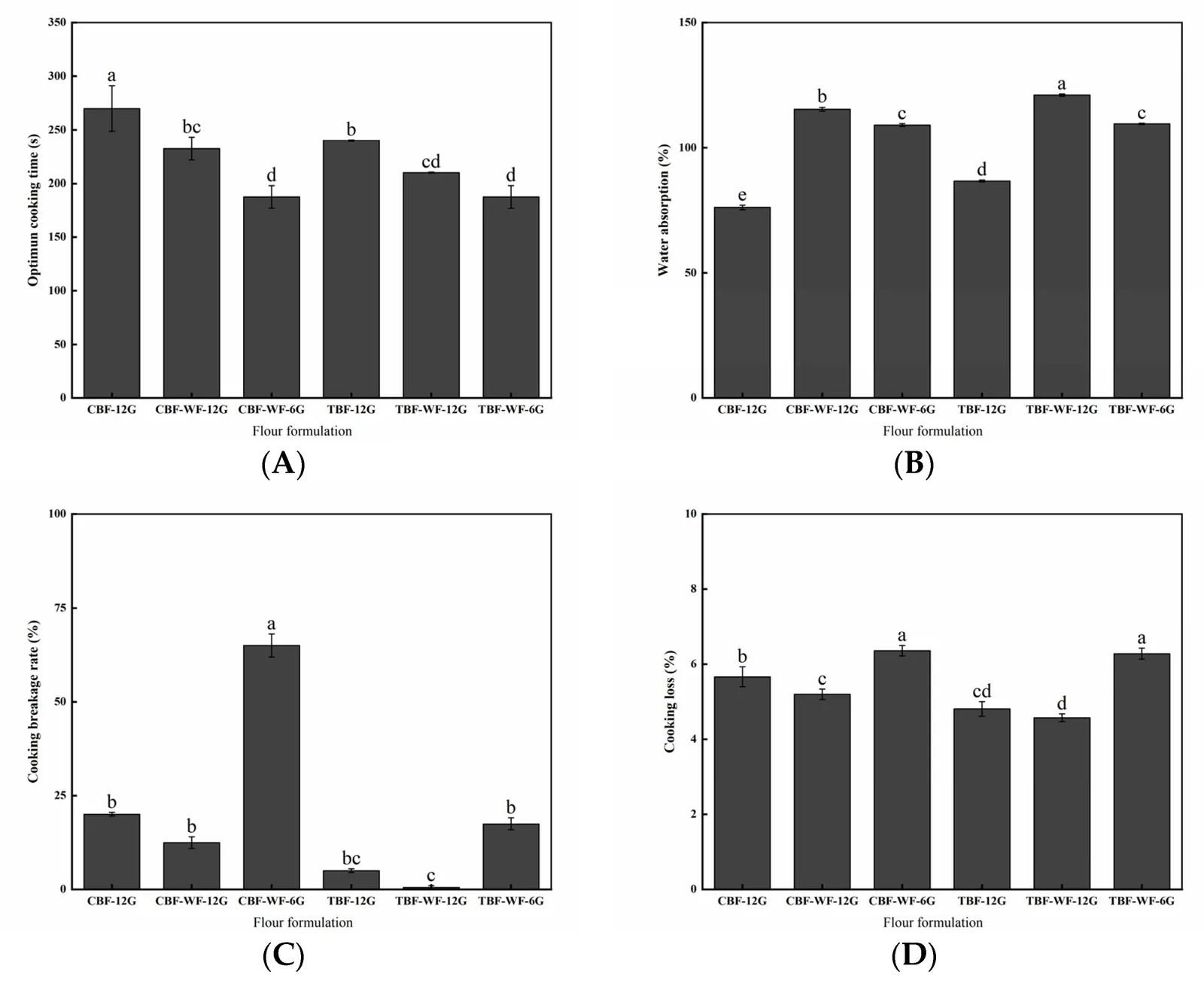
Cooking quality attributes of buckwheat noodles. (**A**) Optimum cooking time. (**B**) Cooking water absorption. (**C**) Cooking breakage rate. (**D**) Cooking loss. For each bar chart, bars with different letters indicate significant differences at *p* < 0.05.

The cooking properties of the six buckwheat noodle formulations, including optimum cooking time, cooking breakage rate, water absorption, and cooking loss, are presented in Figure 4. Optimum cooking time of noodles varied among formulations; for both buckwheat varieties, noodles without added wheat flour required the longest cooking time, while the blend with 6% gluten showed the shortest. Yang et al. [27] found that the optimum cooking time of noodles formulated with gluten and starch rose as the gluten-to-starch ratio increased from 0:100 to 45:55. For both common and Tartary buckwheat-based noodles, the incorporation of wheat flour and gluten markedly increased water absorption of noodles, with the highest value observed in the blend containing 12% added gluten. While high water absorption is generally associated with improved hydration, excessive uptake can compromise noodle firmness and textural integrity [28]; therefore, a moderate range is often preferred. Cooking breakage rate was exceptionally high for the common buckwheat blend with 6% gluten, indicating that insufficient gluten fortification leads to a fragile noodle structure [29]. Cooking breakage rate remained low across all Tartary noodles, reflecting superior structural integrity compared to their common buckwheat counterparts. Cooking loss values were generally slightly higher in wheat flour-fortified samples at 6% gluten addition. Overall, Tartary buckwheat noodles fortified with wheat flour and 12% added gluten exhibited the best combination of high water absorption, low breakage, and acceptable cooking loss, demonstrating promising potential for noodle production.

### 3.6. Texture Attributes of Cooked Buckwheat Noodles

The texture characteristics of cooked buckwheat noodles, including hardness, springiness, chewiness, cohesiveness, and resilience, are summarized in Table 4. Among all formulations, Tartary buckwheat noodles incorporating 50% wheat flour and 12% added gluten (TBF-WF-12G) exhibited the highest values across all parameters, indicating a firm, elastic, and cohesive noodle structure with superior mechanical strength and recovery capacity. This suggests that the increased water absorption during cooking did not adversely affect textural quality of the noodles. The same Tartary buckwheat blend with 6% gluten also showed high hardness and chewiness, though slightly lower than the 12% counterpart. In contrast, buckwheat noodles without wheat flour (both common and Tartary variety) generally displayed lower hardness and chewiness, underscoring the critical role of wheat flour and adequate gluten in forming a robust protein network. Common buckwheat blends with wheat flour and gluten showed improved texture relative to common buckwheat without adding wheat flour but remained inferior to the optimal Tartary-based formulation. Overall, the formulation consisting of 50% Tartary buckwheat, 50% wheat flour, and 12% added gluten was found to yield noodles with optimal texture.

## 4. Conclusions

The incorporation of 50% high-gluten wheat flour together with an appropriate gluten powder effectively improved dough stability of buckwheat dough. All doughs exhibited typical viscoelastic solid-like behavior. For common buckwheat with added wheat flour, the formulation with 6% gluten addition exhibited higher G′ and G″ than that with 12% gluten. For the Tartary blend containing wheat flour, G′ and G″ increased with the level of added gluten. Water was predominantly weakly bound in all doughs, with Tartary doughs showing higher total bound water than common buckwheat doughs. Tartary buckwheat-based flours exhibited substantially higher PV, FV, and SBV than their common buckwheat-based counterparts. The addition of wheat flour and gluten altered the pasting properties of buckwheat noodle flours, and the influence was formulation-dependent. Tartary buckwheat noodles with 50% wheat flour and 12% gluten achieved the best cooking and texture qualities. This study demonstrates the feasibility of improving buckwheat noodle quality by incorporating gluten and wheat flour. However, several limitations deserve further investigation. First, in dough formulation, only a single water addition level was tested for each flour blend, and no other potential ingredients (e.g., emulsifiers, hydrocolloids, or salt) were examined. Second, certain quality attributes, including sensory characteristics, digestibility, and shelf-life, were not assessed. Finally, the noodle microstructure was not characterized, which prevents a full understanding of the property differences among formulations.

## Figures and Tables

**Table 1:** Mixolab characteristic values of different buckwheat flour blends ^†^.

Flour Formulation	WA (%)	DDT (min)	DST (min)
CBF-12G	60.0 ± 0.1 ^b^	5.04 ± 0.16 ^a^	4.77 ± 0.22 ^b^
CBF-WF-12G	60.9 ± 0.2 ^a^	4.85 ± 0.27 ^a^	5.62 ± 0.17 ^a^
CBF-WF-6G	58.1 ± 0.3 ^c^	4.62 ± 0.30 ^a^	5.25 ± 0.32 ^a^
TBF-12G	60.9 ± 0.7 ^a^	2.54 ± 0.33 ^c^	3.45 ± 0.18 ^c^
TBF-WF-12G	58.1 ± 0.1 ^c^	3.58 ± 0.05 ^b^	5.49 ± 0.28 ^a^
TBF-WF-6G	55.4 ± 0.4 ^d^	3.53 ± 0.21 ^b^	5.37 ± 0.34 ^a^

^†^ Values within the same column followed by different lowercase letters are significantly different at *p* < 0.05. WA: water absorption; DDT: dough development time; DST: dough stability time.

**Table 2:** Moisture distributions of dough prepared with different buckwheat flour blends ^†^.

Flour Formulation	Total Bound Water (%)	Free Water (%)
CBF-12G	96.61 ± 0.16 ^d^	3.39 ± 0.16 ^a^
CBF-WF-12G	97.57 ± 0.06 ^c^	2.43 ± 0.06 ^b^
CBF-WF-6G	97.63 ± 0.19 ^c^	2.37 ± 0.19 ^b^
TBF-12G	98.99 ± 0.06 ^a^	1.01 ± 0.06 ^d^
TBF-WF-12G	98.72 ± 0.04 ^b^	1.28 ± 0.04 ^c^
TBF-WF-6G	98.73 ± 0.10 ^b^	1.27 ± 0.10 ^c^

^†^ Values within the same column followed by different lowercase letters are significantly different at *p* < 0.05.

**Table 3:** Pasting property parameters of different buckwheat noodle blends ^†^.

Flour Formulation	PV (cP)	TRV (cP)	BDV (cP)	FV (cP)	SBV (cP)	PT (°C)
CBF-12G	1034.0 ± 18.4 ^d^	1030.5 ± 17.7 ^e^	3.5 ± 0.7 ^d^	1648.5 ± 14.8 ^e^	614.5 ± 36.1 ^e^	94.0 ± 0.6 ^a^
CBF-WF-12G	939.5 ± 14.8 ^e^	925.0 ± 5.7 ^f^	14.5 ± 2.8 ^d^	1618.0 ± 46.7 ^e^	693.0 ± 38.2 ^d^	83.4 ± 5.2 ^ab^
CBF-WF-6G	1139.5 ± 23.3 ^c^	1101.5 ± 21.9 ^d^	38.0 ± 18.4 ^c^	1939.5 ± 0.7 ^d^	838.0 ± 22.6 ^c^	81.5 ± 11.5 ^ab^
TBF-12G	1438.0 ± 18.4 ^a^	1433.5 ± 23.3 ^a^	4.5 ± 4.9 ^d^	2454.5 ± 9.2 ^a^	1016.5 ± 9.2 ^a^	75.9 ± 0.0 ^b^
TBF-WF-12G	1312.0 ± 14.1 ^b^	1225.0 ± 9.9 ^c^	87.0 ± 2.8 ^b^	2064.0 ± 9.8 ^c^	839.0 ± 17.0 ^c^	81.9 ± 2.9 ^ab^
TBF-WF-6G	1464.0 ± 5.7 ^a^	1348.0 ± 4.2 ^b^	116.0 ± 9.9 ^a^	2273.0 ± 25.4 ^b^	925.0 ± 21.2 ^b^	94.9 ± 0.6 ^a^

^†^ Values within the same column followed by different lowercase letters are significantly different at *p* < 0.05. PV: peak viscosity; TRV: trough viscosity; BDV: breakdown viscosity; FV: final viscosity; SBV: setback viscosity; PT: pasting temperature.

**Table 4:** Texture characteristics of cooked buckwheat noodles ^†^.

Flour Formulation	Hardness (g)	Springiness	Chewiness (g)	Cohesiveness	Resilience
CBF-12G	3707.4 ± 209.0 ^d^	0.84 ± 0.08 ^c^	2872.9 ± 170.3 ^c^	0.83 ± 0.02 ^d^	0.61 ± 0.05 ^c^
CBF-WF-12G	3913.1 ± 65.3 ^c^	0.93 ± 0.03 ^ab^	3294.3 ± 148.0 ^b^	0.90 ± 0.02 ^ab^	0.66 ± 0.03 ^b^
CBF-WF-6G	3803.9 ± 141.8 ^cd^	0.87 ± 0.09 ^bc^	2928.9 ± 184.8 ^c^	0.88 ± 0.03 ^bc^	0.64 ± 0.02 ^bc^
TBF-12G	3637.2 ± 129.0 ^d^	0.91 ± 0.04 ^ab^	2975.1 ± 158.5 ^c^	0.87 ± 0.00 ^c^	0.65 ± 0.03 ^bc^
TBF-WF-12G	4522.0 ± 90.5 ^a^	0.96 ± 0.02 ^a^	3731.7 ± 124.0 ^a^	0.92 ± 0.01 ^a^	0.71 ± 0.02 ^a^
TBF-WF-6G	4297.5 ± 170.9 ^b^	0.93 ± 0.02 ^ab^	3649.9 ± 121.6 ^a^	0.89 ± 0.02 ^bc^	0.66 ± 0.05 ^b^

^†^ Values within the same column followed by different lowercase letters are significantly different at *p* < 0.05.

## Data Availability

The original contributions presented in this study are included in the article. Further inquiries can be directed to the corresponding author.
